# Miscellaneous

**Published:** 1891-07

**Authors:** 


### MISCELLANEOUS.

### THE NAIL HIT RIGHT ON THE HEAD.

Rev. Dr. S. B. Rossiter, of the North Presbyterian Church, in a recent sermon, in allusion to the case of Dr. Briggs, answered the question, "What is the matter with our ministers?" in a way that will meet with a general acceptance from all right thinking men. The time has come when religious creeds that will not stand the test of reason and common sense must go by the board. "There is," said he, "a great commotion in the Church these times. It is bubble, bubble, talk and trouble. Presbyterians have a breezy trial on hand, which, if entered upon, will keep the church in convulsions for half a score of years; the Episcopal church is about to try a member for heresy, and at the same time admits a minister from another denomination, who left his own on account of heresy, and this same church is in a state of mind about Phillips Brooks; the Baptist church sees rocks ahead; in the United Presbyterian church there is a family quarrel, and even our friends, the Quakers, are more or less disturbed.

"What is the matter? every one is saying. I think I can only answer the question by saying that some one is revising his personal creed, not only in this country but in all countries. By a strange law that obtains in the minds of people, without communicating much one with another, they weary of this revision and this change, and have become aware that the personal belief of a vast number of people is larger, broader, higher and better than the long accepted formularies of the Church.

"The idea is spreading that we must adapt ourselves to the universal offer of salvation. Opinion, like gunpowder or gun cotton, is the most explosive thing in the world. Press it within narrow limits and you invite explosion, and we are liable to be blown all to pieces. Are we to be alarmed, then? No. We should rejoice at the diversity of opinion, looking at Dr. Briggs, Phillips Brooks, Dr. Newton and Dr. Bridgman as the bright spots in the explosion, indicating the center of vigor and motion in a great movement."

### ELEPHANTINE INTELLIGENCE.

One evening, soon after my arrival in Eastern Assam, and while the five elephants were being fed opposite the bungalow, says an eastern traveler, I observed a young and lately caught one step up to a bamboo fence and quietly pull up one of the stakes. Placing it under its foot it broke off a piece with its trunk, and after lifting it to its mouth threw it away. It repeated this twice or thrice, and then drew another stake. Seeing that the bamboo was old and dry, I asked the reason for this, and was told to wait and see what the elephant would do. At last he seemed to get a piece that suited him, and holding it in the trunk firmly, and stepping the left fore-leg well forward, it passed the piece of bamboo under the arm-pit, so to speak, and began to scratch with some force. My surprise reached its climax when I saw a large elephant leech fall to the ground, quite six inches long and thick as one's finger, and which, from its position, could not be easily detached without this scraper which was deliberately made by the elephant. On another occasion, when traveling at a time of the year when large flies are so tormenting to an elephant, I noticed that the one I rode had no fan or wisp to beat them off with. The driver, at my order, slackened pace and allowed her to go to the side of the road, when for some moments she moved along, rummaging the smaller jungle on the banks. At last she came to a cluster of young shoots well branched, and after feeling among them and selecting one, raised her trunk and neatly stripped down the stem, taking off all the lower branches, and leaving a fine bunch on top. She deliberately cleaned it down several times, and then laying hold at the lower end broke off a beautiful fan or switch about five feet long, handle included. With this she kept the flies at bay, flapping them off on each side. Say what we may, these are both really *bona-fide* instruments, each intelligently made for a definite purpose.

### WALKING LEAF AND WALKING STICK.

Stanly Wood's *Great Divide* says: "Who ever heard of green leaves falling from a tree, and, after lying on the ground a few minutes, crawling toward the trunk of the tree, ascend it, and resume their former position?" and then proceeds to depict the surprise of some English sailors on an island near Australia when they first discovered the wonderful walking leaf. There was no occasion to go to

Australia to find these insects, as they are met with frequently right here in the United States. The walking stick is another peculiar form of insect life common to America, which received its name from its close resemblance to a twig. Different species of this insect resemble twigs from different kinds of trees.

---

### A TRUTH WORTH CONSIDERING.

The highest spiritual power can only act through like power within our own being and to the extent of its unfoldment. The most potent angel cannot save from destruction, on the material plane, if there is nothing within the individual to relate him to the celestial spheres. We are self-grown and self-saved. We are not independent until we become self-dependent. All the experiences of life are for the purpose of teaching us to be self-dependent. We are the weaker when we rely upon others to do that we should do ourselves.— *World's Advance Thought.*

---

### ANTICS OF A BABY RHINOCEROS.

Mr. Stanley gives the following account of a baby rhinoceros, which his Nubians brought into camp. "We tied the baby, which was as large as a prize boar, to a tree, and he fully showed what combativeness there was in his nature. Sometimes he mistook the tree for an enemy, and rushed to the attack, battering it with his horny nose until, perceiving that the tree obstinately resisted him, he would halt to reconnoitre it, as though he had the intention of assaulting it by another method ; but at such times some wicked Zanzibari boys prodded him in the hams with a reed cane, and, uttering a startling squeal of rage, he would dash at the offenders to the length of his tether. He seemed to me to be the stupidest, most ireful, intractable little beastie that ever I had met. Feeling himself restrained by the cord, he felt sure that it must be the tree that was teasing him, and he would make another dash at it with such vehemence as to send him on his haunches ; prodded, pricked in the rear, he squealed again, and, swinging round with wonderful activity, he would start headlong, to be flung on his back by the rope, until at last, feeling that it would be only misery to him to be carried to the coast, he was consigned to the butcher and his assistants."

---

### LOOK OUT FOR THE "RUSSIAN ITCH."

The large number of Russian Hebrews flocking into Hamburg for emigration to America is said to be the cause of a peculiar distemper that has appeared in that vicinity, known as the "Russian itch."

Colonel John B. Weber, the superintendent of the Immigration Bureau, yesterday wrote to the agents of the Hamburg Packet Company, stating that under the new immigration law he believed that the "Russian itch" was a loathsome disease, and that if immigrants were brought here suffering from it they would be returned at the expense of the company.

